# Association between hearing organ and renal function in young adult type 1 diabetic patients: A cross-sectional study

**DOI:** 10.1038/s41598-018-31057-6

**Published:** 2018-08-23

**Authors:** Mariusz Dąbrowski, Grażyna Mielnik-Niedzielska, Andrzej Nowakowski

**Affiliations:** 1 University of Rzeszów, Faculty of Medicine, Institute of Nursing and Health Sciences, Rzeszów, Poland; 2 Diabetic Outpatient Clinic, Medical Center NZOZ “Beta-Med”, Rzeszów, Poland; 3 Medical University of Lublin, Department of Pediatric Otolaryngology, Phoniatry and Audiology, Lublin, Poland; 4 Medical University of Lublin, Professor emeritus, Lublin, Poland

## Abstract

Type 1 diabetes can lead to impaired function of many organs and tissues. The aim of this study was to evaluate associations between hearing and kidney function in young adult type 1 diabetic patients. 31 patients (9 women) with type 1 diabetes, aged <45, with disease duration <10 years were included. Blood and urine samples for laboratory tests and urinary albumin excretion (UAE) assessment were obtained. eGFR was calculated with CKD-EPI formula. In all patients pure-tone audiometry, transient evoked otoacoustic emissions and auditory brainstem responses were evaluated, also eye fundus was examined. Mean patients’ age was 29.5 ± 7.0 years and disease duration 4.6 ± 2.6 years. All patients had eGFR > 60.0 ml/min/1.73 m^2^. In one case microalbuminuria and in 3 patients early retinopathy were revealed. Linear correlation between eGFR and hearing threshold at 4, 6, 8 and 12 kHz was found. Patients with hearing impairment (n = 7) had lower eGFR 108.8 vs. 121.7 ml/min/1.73 m^2^, p = 0.047 compared to normal-hearing subjects. Also patients with absence of otoacoustic emissions in at least one ear had lower eGFR, 103.1 vs. 123.3 ml/min/1.73 m^2^, p < 0.001, compared to the remaining group. In auditory brainstem responses we found significant linear correlation between eGFR and wave III and interval I-III latencies, and between UAE and waves III, V and interval I-III latencies. This study suggests existence of relationship between hearing and kidney function in type 1 diabetic patients. Pathways directly linking hearing and renal function are unknown. Larger studies are necessary to further analyze these relationships.

## Introduction

Incidence of type 1 diabetes continues to rise. In Podkarpackie Region in South-Eastern Poland, in population below 19 it reached almost 20 new cases per 100,000 children and adolescents in this age range in the year 2013 (Malesa and Dąbrowski, unpublished data). However, type 1 diabetes can be diagnosed also in population above 30. In United Kingdom 42% of new cases of type 1 diabetes were diagnosed in this age group^1^. Prevalence of type 1 diabetes mellitus in high income countries is estimated to consist 7–12% of all diabetes cases^2^. Poorly controlled type 1 diabetes can lead to development of a long-term diabetic complications. Diabetic retinopathy, nephropathy, neuropathy and cardiovascular disease are the most widely known consequences of diabetes^3^. However, type 1 diabetes can also affect function of other organs. Dysfunction of hearing organ is significantly more frequent in type 1 diabetic patients, compared to non-diabetic population, which was summarized in a recently published meta-analysis^4^. Abnormalities in hearing function, also subclinical, can be found even in a young adults with short-lasting diabetes, which was documented in our earlier published paper^5^.

The hearing function can be assessed using several audiological tests. The pure-tone audiometry (PTA) is the most common test used to evaluate hearing thresholds at different frequencies, which reflects the function of both peripheral and central auditory pathway^6^. Otoacoustic emissions (OAEs) evaluation is used to determine cochlear micromechanics status, especially outer hair cells (OHC) function. The cochlear response is evoked by a click or pure-tone stimulus and its amplitude is expressed in dB. The higher is the amplitude of OAE’s, the better is the OHC function^7^. Evaluation of the auditory brainstem response (ABR) is the most commonly used non-invasive electrophysiological test to determine function of the retrocochlear part of the auditory pathway, up to the brainstem level^8^.

Diabetic kidney disease is one of the most common diabetic complications, which can finally lead to the end-stage renal disease (ESRD) requiring renal replacement therapy. Prevalence of ESRD in type 1 diabetes in Finland after 20 and 30 years of disease duration was 2.2% and 7.0% of patients respectively^9^. In Norway it was even less prevalent in patients with childhood-onset type 1 diabetes: 0.7%, 2.9% and 5.3% after respectively 20, 30 and 40 years of type 1 diabetes duration^10^. Higher ESRD was observed in the Pittsburgh Epidemiology of Diabetes Complications (EDC) study with 5.5%, 14.5% and 26.5% affected after 20, 30 and 40 years of diabetes duration respectively^11^. Usually deterioration of kidney function was considered to be preceded by increased urinary albumin excretion (UAE). However, progressive decline of kidney function measured by estimated glomerular filtration rate (eGFR) is observed also in patients without albuminuria. And inversely, not always patients with overt proteinuria have progressive renal decline^12^. Diabetic complications frequently correlate to each other and, regardless of stage, diabetic nephropathy is almost exclusively seen in patients with co-existing diabetic retinopathy^13^.

The primary objective of our cross-sectional study, was to evaluate whether auditory organ function, assessed by PTA, OAEs and ABR correlates to kidney function in a group of relatively young adult type 1 diabetic subjects with a short duration of the disease. The secondary objective was to evaluate other relationships between analyzed variables including lipid profile, blood pressure and presence of retinopathy. This study is a post-hoc analysis of our previously published data^5,14,15^.

## Results

The mean age of the study participants was 29.5 ± 7.0 years and mean type 1 diabetes duration 4.6 ± 2.6 years. In 3 patients eGFR was between 75.0 and 90.0 mL/min/1.73 m^2^, the rest had eGFR above 90.0 mL/min/1.73 m^2^. In one case UAE within microalbuminuria range was revealed (43.3 mg/L), however, in this patient urinary albumin/creatinine ratio (UACR) was <30 mg/g. In 3 patients early background retinopathy was found. Full characteristics of study participants are presented in Table 1.

**Table 1:** Characteristics of the study participants.

Parameter	Mean	Standard Deviation	Range
Creatinine (μmol/L)	66.30	17.68	35.36–106.08
eGFR CKD-EPI (mL/min/1.73 m^2^)	118.75	21.44	79–167.4
Age (years)	29.52	6.96	18–43
Gender			
Male, n (%)	22 (71.0%)	N/A	N/A
Female, n (%)	9 (29.0%)		
Diabetes duration (months)	55.7	31.3	3–117
HbA_1c_ (mmol/mol)	61.8	16.6	31.1–109.8
HbA_1c_ (%)	7.80	1.50	5–12.2
Urinary albumin excretion (mg/L)	9.51	7.88	5–43.3
Total cholesterol (mmol/L)	4.67	1.02	2.72–7.17
HDL-cholesterol (mmol/L)	1.50	0.38	0.62–2.23
LDL-cholesterol (mmol/L)	2.67	0.98	0.57–4.97
Triglycerides (mmol/L)	1.03	0.77	0.38–3.60
Retinopathy, n (%)	3 (9.7%)	N/A	N/A
SBP (mm Hg)	130.4	11.9	104–156
DBP (mm Hg)	75.5	7.2	62–99
Hypertension, n (%)	8 (25.8%)	N/A	N/A
BMI (kg/m^2^)	23.05	3.36	18.6–34.1
Hearing impairment, n (%)	7 (22.6%)	N/A	N/A
Mean TEOAE amplitude at 1.2–3.5 kHz (dB)	7.74	4.43	0.1–20.3
TEOAE absent, n (%)	7 (22.6%)	N/A	N/A
Wave I latency (ms)	1.73	0.12	1.55–2.00
Wave III latency (ms)	3.91	0.19	3.50–4.35
Wave V latency (ms)	5.78	0.25	5.10–6.30
Interval I-III latency (ms)	2.17	0.16	1.80–2.50
Interval III-V latency (ms)	1.85	0.16	1.45–2.10
Interval I-V latency (ms)	4.03	0.20	3.55–4.40

**Figure 1:**
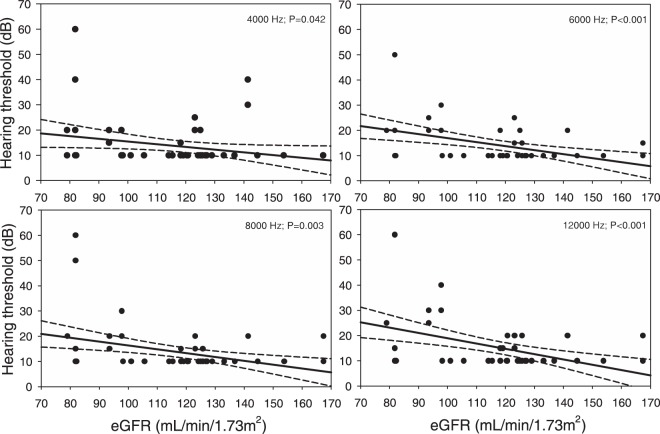
Linear correlation between eGFR and hearing thresholds at 4000 Hz, 6000 Hz, 8000 Hz and 12,000 Hz (scatter plot, regression lines and 95% confidence intervals).

In PTA mild to moderate bilateral hearing impairment was found in 7 patients. These subjects had significantly lower eGFR compared to the patients with normal hearing, 108.8 ± 26.4 vs. 121.7 ± 19.1 mL/min/1.73 m^2^, p = 0.047. However, they were also significantly older, 35.0 ± 7.8 vs. 27.9 ± 6.0 years respectively, P = 0.016. A significant negative linear correlation between eGFR and hearing threshold was revealed at frequencies 4 kHz (r = −0.259, P = 0.042), 6 kHz (r = −0.411, P < 0.001), 8 kHz (r = −0.373, P = 0.003) and 12 kHz (r = −0.431, P < 0.001) (Fig. 1). Also other variables: age, HDL and LDL cholesterol, triglycerides and BMI demonstrated linear correlation with hearing threshold in these frequencies. After all these variables were included in the multiple linear regression analysis, the only variables significantly positively correlated with elevated hearing threshold in all frequencies were age and LDL cholesterol, HDL cholesterol was inversely associated with hearing threshold in 4 kHz and triglycerides showed positive relationship with hearing threshold in 6 kHz and 12 kHz. When age was excluded from the analysis, eGFR appeared to be significantly associated with hearing threshold at 6 kHz (P = 0.003), 8 kHz (P = 0.008) and 12 kHz (P = 0.002). Also LDL cholesterol and triglycerides revealed positive linear correlation with hearing threshold at these frequencies. No association between hearing threshold and UAE was found.

In TEOAE evaluation absence of TEOAE in at least one ear was revealed in 7 patients. These subjects, compared to patients with preserved TEOAE, had significantly lower eGFR (103.1 ± 17.0 vs. 123.3 ± 20.6 mL/min/1.73 m^2^), P < 0.001. No relationship between UAE and lack of TEOAE was found, also no significant difference in age between these two groups was revealed. No linear correlation between eGFR and TEOAE amplitude was revealed, while an insignificant trend towards negative linear correlation between UAE and TEOAE amplitude was observed (r = −0.230, P = 0.077).

**Figure 2:**
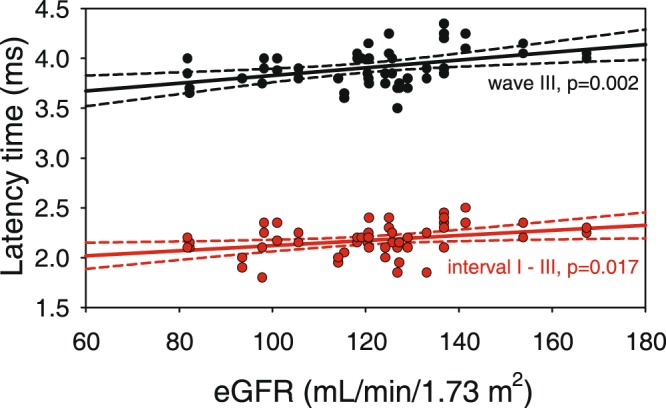
Linear correlation between eGFR and wave III and interval I-III latencies (scatter plot, regression lines and 95% confidence intervals).

**Figure 3:**
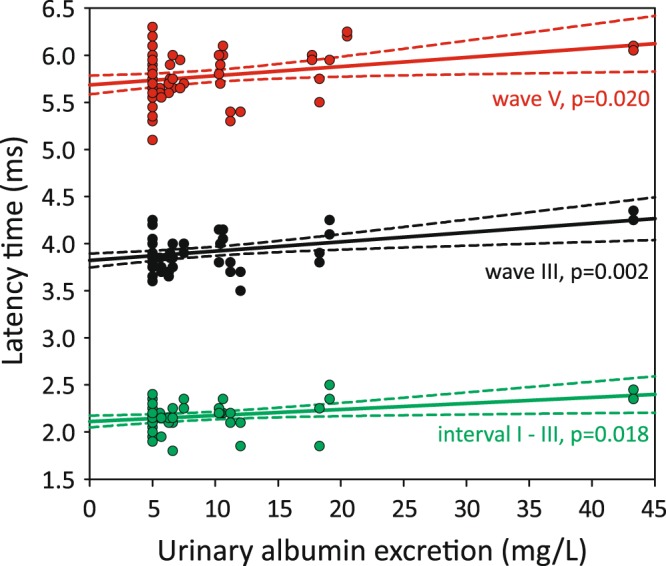
Linear correlation between urinary albumin excretion and wave III, wave V and interval I-III latencies (scatter plot, regression lines and 95% confidence intervals).

In ABR significant positive linear correlation of eGFR with wave III latency (r = 0.406, P = 0.002) and interval I-III (r = 0.324, P = 0.017) was revealed (Fig. 2). Also positive linear correlation between UAE and latencies of wave III (r = 0.415, P = 0.002), wave V (r = 0.299, P = 0.020) and interval I-III (r = 0.328, P = 0.018) was found (Fig. 3). Among other variables only systolic blood pressure (SBP) demonstrated positive linear correlation with wave III latency. After adjustment for SBP association between both eGFR as well as UAE and wave III latency remained significant (r = 0.308, P = 0.029 and r = 0.315, P = 0.005 respectively).

Despite very low number of patients with retinopathy, eGFR in these subjects was significantly lower compared to patients without retinopathy, 102.8 ± 32.5 vs. 122.0 ± 18.2 mL/min/1.73 m^2^), p = 0.028. Retinopathy was also associated with presence of hearing impairment, P = 0.002 and with significantly higher hearing threshold at frequencies 1 kHz (P < 0.001), 2 kHz (P = 0.008) and 4 kHz (P = 0.030).

UAE demonstrated strong linear correlation with HbA_1c_ level, r = 0.571, P < 0.001. No relationship between HbA_1c_ and eGFR or retinopathy was found.

Lipid parameters, blood pressure values and BMI were significantly associated with abnormalities in the audiological evaluations, which was described previously elsewhere^15^. Significantly lower HDL cholesterol level (1.00 ± 0.33 vs. 1.53 ± 0.33 mmol/L, P = 0.014), and significantly higher triglycerides (2.58 ± 0.91 vs. 0.88 ± 0.55 mmol/L, P = 0.012) were also revealed in patients with retinopathy compared to subjects with normal eye fundus. No association was found between serum lipids, blood pressure or BMI and eGFR or UAE.

## Discussion

Sense of hearing is one of the most important tools of social communication. Hearing impairment assessed in PTA is significantly more prevalent among patients both with type 1 and type 2 diabetes, compared to non-diabetic population^4,16^. Abnormalities in hearing organ function are also seen in OAEs and ABR evaluations^4,5,14,15^. Apart of age, among factors leading to impaired hearing function role of blood pressure, lipid abnormalities and elevated BMI are pointed out^15,17,18^.

Data regarding associations between hearing impairment and kidney function in diabetic patients are scarce. Bainbridge *et al*. in their study on Hispanic/Latino population found significantly higher prevalence of hearing impairment in adult diabetic patients with moderate kidney impairment compared to subjects with normal eGFR^19^. In a large cross-sectional study conducted among 5,226 Korean adults, hearing impairment was strongly associated with eGFR < 60 mL/min/1.73 m^2^, systolic and diastolic blood pressure, while no association with microalbuminuria, diabetes, HbA_1c_, hypertension or dyslipidemia was revealed^20^. Also in another population-based study including 2,564 participants moderately decreased kidney function was associated with hearing impairment^21^.

Our study suggests the existence of relationship between hearing and renal function in young adult type 1 diabetic patients. Mild to moderate hearing impairment was associated with lower eGFR at subclinical level (only 3 subjects had eGFR < 90 mL/min/1.73 m^2^ and none of them had eGFR < 75 mL/min/1.73 m^2^). Also positive linear correlation between eGFR and hearing threshold at high frequencies was revealed (although mean value was within normal range). However, after adjustment for age, lipid parameters and BMI this relationship lost its significance. Interestingly, similarly to Korean study^20^, no association between hearing impairment and microalbuminuria was found. We also did not find significant relationship between hearing thresholds at different frequencies and microalbuminuria.

Association between OAEs and renal function in type 1 diabetic patients was analyzed in only one study. Hou *et al*. did not reveal relationship between DPOAE and/or TEOAE with creatinine or albuminuria level^22^. We revealed significantly lower eGFR (but still within normal range) in patients with a lack of TEOAEs. The amplitude of OAEs tended to correlate with the level of microalbuminuria, but this relationship did not attain the level of statistical significance.

Correlation between ABR and renal function in diabetic patients was also analyzed by Hou *et al*.^22^. They revealed significant relationship between ABR abnormalities and albuminuria but not with creatinine level. We also found association between UAE and ABR results: the higher was UAE, the longer was conduction time through auditory pathway (positive linear correlation of UAE with wave III, V and interval I-III latencies). Interestingly, we found unexpected association between eGFR and ABR results – the higher (i.e. better) was eGFR, the longer (i.e. worse) was conduction time through the distal part of the auditory pathway (positive linear correlation of eGFR with wave III and interval I-III latencies).

Many genetic, metabolic, hemodynamic, environmental and inflammatory factors are involved in development of diabetic complications. Some of them are modifiable, while other are non-modifiable. Many of them are common for both hearing impairment and diabetic kidney disease. Lipid abnormalities, elevated blood pressure and increased BMI are considered to be modifiable factors related to impaired hearing organ function in diabetes. Data regarding HbA_1c_ are divergent^4,5,14,15,18–20,22–24^. In diabetic kidney disease detrimental role of poor metabolic control is clear and also other mentioned above factors play a role in diabetic nephropathy development^23^. In our study, as it could be expected, microalbuminuria was strongly associated with HbA_1c_ level and with longer conduction time in auditory pathway (which indicate existence of central auditory neuropathy). However, UAE was not associated neither with hearing threshold, nor with lack of TEOAE. Inversely, lower eGFR was associated with the presence of hearing impairment, higher hearing threshold at high frequencies and with lack of TEOAE, while, unexpectedly, in ABR lower eGFR was associated with shorter conduction time in auditory pathway. These discrepancies are in fact not easy to explain and require further investigation. Nevertheless, they indicate possible different pathogenic pathways linking hearing organ function with albuminuria level and excretory kidney function measured by eGFR. Moreover, it is worth noting that all associations revealed in our study were present at sub-clinical level and none of the study participants had an overt impaired kidney function.

The main limitation of our study is a small number of participants, thus a random effect of our findings cannot be totally excluded. Another limitation is its cross-sectional design, which did not allow us to find causal relationship between analyzed variables. And finally, eGFR results calculated using CKD-EPI_cr_ formula can be influenced by body composition, e.g. high muscle mass. Nevertheless, our findings suggest the existence of association between hearing organ and kidney function in young adult type 1 diabetic patients with short duration of the disease. Obviously, larger, prospective studies are necessary to further analyze this relationship, to identify factors and pathogenic pathways directly linking these two complications of diabetes, and to determine whether they occur simultaneously or one of them predicts another.

## Methods

The study group consisted of 31 patients (9 women) with type 1 diabetes. The inclusion criteria were: age below 45 (to avoid age-related hearing impairment) and diabetes duration less than 10 years (to avoid advanced diabetic complications). The exclusion criteria were: type 2 diabetes, clinically overt hearing impairment, occupational noise exposure or a history of ototoxic medications use.

In all subjects body weight and height were measured and body mass index (BMI) was calculated. Blood pressure was measured in a sitting position after at least 5 minutes of rest using the automatic Omron 705 IT blood pressure monitor (Omron Healthcare Europe BV, Hoofddorp, The Netherlands). Then fasting venous blood samples for laboratory tests (lipid profile and creatinine level) were obtained. They were measured in the certified laboratory using an Architect c8000 analyzer (Abbott Laboratories, Irving, TX, USA). HbA_1c_ level from capillary blood sample and urinary albumin excretion (UAE) from the first morning urine sample were measured using certified DCA 2000® + analyzer (Siemens, Elkhart, IN, USA) with the monoclonal antibody method. To calculate eGFR Chronic Kidney Disease Epidemiology Collaboration (CKD-EPI_cr_) equation, currently recommended by Diabetes Poland was used^25,26^:

$$\begin{array}{rcl}{\rm{GFR}} & = & {\rm{141}}\,\ast \,{\rm{\min }}\,{(\mathrm{Scr}/{\rm{\kappa }},1)}^{{\rm{\alpha }}}\,\ast \,{\rm{\max }}\,{(\mathrm{Scr}/{\rm{\kappa }},1)}^{-{\rm{1.209}}}\\  &  & \,\ast \,{\rm{0}}{{\rm{.993}}}^{{\rm{Age}}}\ast \,{\rm{1.018}}\,[{\rm{if}}\,{\rm{female}}]\ast 1{\rm{.159}}\,[{\rm{if}}\,{\rm{black}}]\end{array}$$

Scr is serum creatinine (mg/dL), κ is 0.7 for females and 0.9 for males, α is −0.329 for females and −0.411 for males, min indicates the minimum of Scr/κ or 1, and max indicates the maximum of Scr/κ or 1.

Then patients were referred to the audiological laboratory in the Otorhinolaryngology Department at the Regional Specialist Hospital in Rzeszów, Poland. After a detailed ENT examination by the same ENT specialist (to exclude external and middle ear abnormalities), audiological tests were performed.

In all patients pure-tone audiometry, transient evoked otoacoustic emissions (TEOAE) and auditory brainstem responses were evaluated. Detailed methodology of audiological tests was described elsewhere^5^. Mild and moderate hearing impairment were recognized at hearing thresholds exceeding 20 and 40 dB respectively, in at least one frequency^27^. The mean TEOAE amplitude below 6 dB in a band range 1.2–3.5 kHz was considered as a lack of otoacoustic emission^28^.

In all but one patient eye fundus examination by an experienced ophthalmologist was performed within 3 months window from audiological evaluations.

Statistical analysis of the data was performed using SigmaPlot for Windows, version 12.5 (Systat Software Inc., San Jose, CA, USA). The continuous data are presented as mean ± SD (standard deviation). Differences between two groups were analyzed using a Student’s t-test or by a Mann-Whitney rank sum test where appropriate. The categorical data are presented as numbers and they were compared using χ^2^ test. The linear correlations between analyzed variables were assessed using Pearson product moment correlation test after checking the normality of distribution by Shapiro-Wilk test. To assess the strength and independency of these associations, the multiple linear regression test was used. A P value of < 0.05 was considered statistically significant.

### Ethical approval and informed consent

The study was approved by the Bioethics Committee at the District Medical Chamber in Rzeszów and by the all appropriate administrative bodies. The study was conducted in accordance with ethical standards laid down in an appropriate version of the Declaration of Helsinki, and in Polish national regulations. All subjects signed informed consent form.
